# Microfluidic Collective Cell Migration Assay for Study of Endothelial Cell Proliferation and Migration under Combinations of Oxygen Gradients, Tensions, and Drug Treatments

**DOI:** 10.1038/s41598-019-44594-5

**Published:** 2019-06-03

**Authors:** Hsiu-Chen Shih, Tse-Ang Lee, Hsiao-Mei Wu, Ping-Liang Ko, Wei-Hao Liao, Yi-Chung Tung

**Affiliations:** 1 Research Center for Applied Sciences, Academia Sinica, 128 Academia Rd. Sec. 2, Taipei, 11529 Taiwan; 2 College of Engineering, Chang-Gung University, Taoyuan, 33302 Taiwan

**Keywords:** Cell migration, Assay systems

## Abstract

Proliferation and migration of endothelial cells play an important role in many biological activities, and they can be regulated by various microenvironmental factors. In this paper, a novel microfluidic collective cell migration assay is developed to study endothelial cell migration and proliferation under combinations of three oxygen conditions: normoxia, oxygen gradient, and hypoxia and three medium compositions: normal growth medium, the medium with cytochalasin-D for actin polymerization inhibition, and with YC-1 for hypoxia-inducible factor (HIF) inhibition. The microfluidic device designed in the paper allows cell patterns formed with consistent dimensions using laminar flow patterning. In addition, stable oxygen gradients can be generated within the device by a spatially confined chemical reaction method. The device can be operated in conventional cell incubators with minimal chemical reagents and instrumentation for practical applications. The results show directional collective cell migration of the endothelial cells under the oxygen gradients for all the medium compositions. The directional behavior has never been discussed before, and indicates critical roles of oxygen gradients in guiding endothelial cell migration during various biological activities. The developed assay provides a practical yet powerful tool for further i*n vitro* study of endothelial cell behaviors under various physiological microenvironments.

## Introduction

Proliferation and migration of endothelial cells lining the inner surface of blood vessels play critical roles during various physiological development and disease progression processes. For example, during angiogenesis, formation of new blood vessels from existing ones, endothelial cells migrate at the forefront and proliferate. The proliferation and migration are highly regulated by various cellular microenvironmental factors including chemical and physical ones^1–3^. For example, researchers have discovered that vascular endothelial growth factor (VEGF) plays a key role in physiological angiogenesis during embryogenesis, skeletal growth and reproductive functions^1^. In addition, Song *et al*. have investigated effects of various fluidic forces on endothelial cell sprouting^4^.

Among various factors, oxygen tension is one of the most essential factors regulating the angiogenesis process^5^. As early as 1983, Knighton *et al*. have found that oxygen tension regulates the expression of active angiogenesis factor by macrophages and affects the wound healing speed^6^. A number of studies have also discussed the roles of oxygen tension in regulating the endothelial cell behaviors. For instance, Zhao *et al*. showed that low-oxygen (5%) pretreatment enhances endothelial cell growth and retention under shear stress^7^. Michiels *et al*. discussed the responses of endothelial cells in the aspect of gene expression, cytokine and growth factor secretion after exposure to acute and longer period hypoxia^8^.

However, existing researches focus on how uniform oxygen tension affects endothelial cells and their surrounding cells for cytokine secretion and further regulates endothelial cell proliferation and migration. Few research studies the direct effects of spatial oxygen distribution, and combinations of oxygen conditions and drug treatments on endothelial cell behaviors due to technical limitations, especially the capability of generating stable oxygen gradients for cell culture. Recently, microfluidics has been broadly exploited for biomedical studies due to its advantageous properties^9,10^. Microfluidics provides great controllability in cellular microenvironments with less cell samples and reagent consumption. Consequently, various microfluidic cell culture devices capable of controlling microenvironments have been developed for *in vitro* cell studies^11–14^.

In this paper, we design a microfluidic collective cell migration assay to study the endothelial cell proliferation and migration under various combinations of oxygen tensions, gradients, and drug treatments. The laminar flow pattern within the microfluidic devices provides a reliable scheme to generate cell patterns with consistent dimensions^15,16^. In addition, the spatially confined chemical reaction method is exploited to generate stable oxygen gradients within the microfluidic device for the collective cell migration assay study^17–19^. The device provides a convenient cell culture platform for oxygen gradient generation without using bulky gas cylinders, tedious interconnections, and is compatible to conventional cell incubators for optimal culture conditions. Furthermore, the oxygen gradients can be adjusted by varying concentration of chemical reactants and device geometries^20^.

In the experiments, human umbilical vein cells (HUVECs) are exploited for the collective cell migration assays using the microfluidic devices. Collective cell migration assays with combinations of three different oxygen conditions: normoxia, oxygen gradient, and uniform hypoxia (1%), and three medium compositions are conducted in the experiments. In the experiments, two drugs, cytochalasin-D and YC-1 (3-(5′-hydroxymethyl-2′-furyl)-1-benzylindazole) are tested to study their effects on the HUVECs collective cell migration. Cytochalasin-D is a cell membrane permeable fungal toxin that has been found to bind to F-actin polymer and prevent polymerization of actin monomers; therefore, it can be used as a potent actin polymerization inhibitor^21,22^. YC-1 is a hypoxia-inducible factor (HIF) inhibitor that down regulates HIF-1α and HIF-2α at the post-translational level, and it has been developed as a novel anti-cancer drug^23,24^. The collective cell migration speeds and cell numbers are analyzed based on the images collected during the experiments. The microfluidic collective cell migration assays of endothelial cells under various oxygen conditions and drug treatments can provide biologists insightful information and fundamental understanding of their responses under physiological microenvironments during various biological activities.

## Materials and Methods

### Device design and fabrication

**Figure 1:**
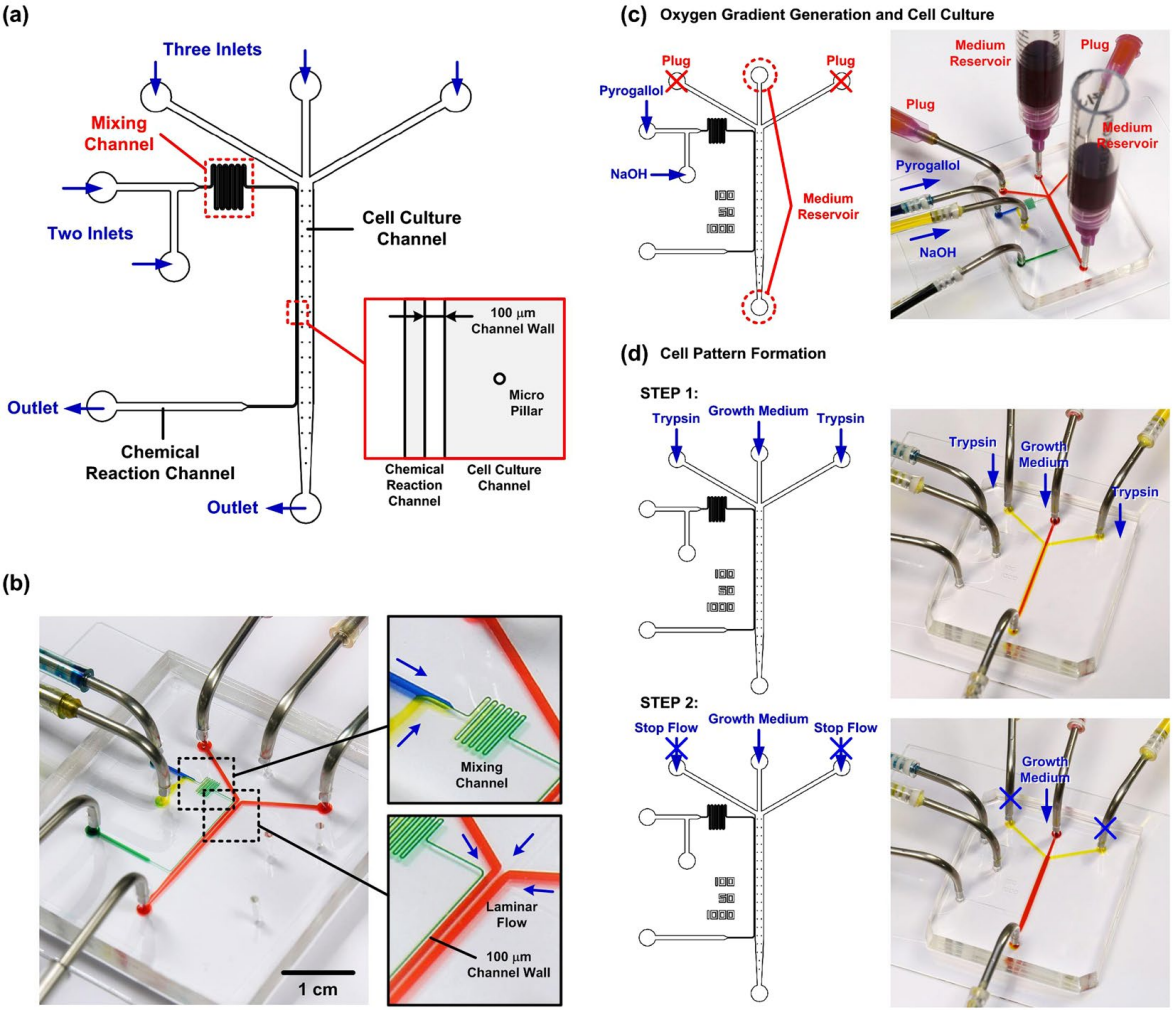
(**a**) Schematic of the microfluidic device capable of performing collective cell migration assays with oxygen gradients. (**b**) Experimental photos of the fabricated microfluidic devices. (**c**,**d**) Operation of the microfluidic devices for oxygen gradients generation and cell pattern formation.

The microfluidic device is constructed using an elastomeric material, polydimethylsliloxane (PDMS), due to its optical transparency, manufacturability, and gas permeability^25^. The device is composed of two sets of channels as shown in Fig. 1(a). A cell culture channel, designed with three inlets and one outlet, is used to culture cells, form cell patterns, and perform collective cell migration assays.

The cell pattern is generated using laminar flow patterns with growth medium and trypsin^26^. A chemical reaction channel is exploited for an oxygen scavenging chemical reaction to generate oxygen gradients inside the cell culture channel^17^. The channel is designed with two inlets and one outlet to spatially control the chemical reaction at the desired location for the oxygen gradient generation. The chemical reactants are introduced into the channel from the different inlets, and start to mix and react with each other in a meander-shaped mixing channel right before flowing into the area next to the cell culture channel. The cell culture channel and the chemical reaction channel are separated by a thin wall with width of 100 µm. Due to high diffusivity of oxygen within PDMS, oxygen can diffuse through the channel wall to alter oxygen tensions inside the cell culture channel without direct contact between the cell culture medium and the chemical reactants. The device architecture provides an efficient method to generate oxygen gradients in micro-scale for cell culture with minimum chemical consumption and great cell incubator compatibility^17–19^.

The device is fabricated using the well-developed soft lithography replica molding technique. In brief, the master mold is prepared by patterning a layer of negative tone photoresist (SU-8 2050, MicroChem Co., Newton, MA) with channel features on a silicon wafer using conventional optical lithography processes. PDMS pre-polymer with 1:10 (w/w) curing agent to base ratio is then poured on the mold, and cured at 60 °C for more than 4 hours. The PDMS layer is then irreversibly bonded to a bottom layer, which is prepared by spin coating the aforementioned pre-polymer on a glass slide and curing. The bonding is achieved using oxygen plasma surface treatment at power of 90 W with 250 mTorr O_2_ gas on both layers for 40 seconds in a plasma treatment system (PX-250, Nordson MARCH, Concord, CA). Figure 1(b) shows experimental photos of a fabricated microfluidic device filled with food dyes. Syringe pumps are exploited to accurately control the flows inside the fabricated device.

### Generation and characterization of oxygen gradients

In order to generate oxygen gradients inside the cell culture channel, an oxygen scavenging chemical reaction is performed in the chemical reaction channel. In the experiments, pyrogallol (benzene-1,2,3-triol, C_6_H_6_O_3_) (87-66-1, Alfa Aesar, Ward Hill, MA) and NaOH (30620, Sigma-Aldrich, St Louis, MO) are exploited for the reaction. When pyrogallol in alkaline solution, it absorbs oxygen from the surrounding rapidly^27^. In the experiments, the oxygen gradients are generated by flowing pyrogallol with concentration of 100 mg/ml and 1M NaOH into the chemical reaction channel from the different inlets with flow rates of 10 *µ*l/min as shown in Fig. 1(c). To characterize the oxygen gradient established in the middle cell culture channel, an oxygen sensitive fluorescence dye, tris (2,2′-bipyridyl) ruthenium (II) chloride, hexahydrate (50525-27-4, Acros Organics, Geel, Belgium) is exploited to flow into the cell culture channel. The fluorescence lifetime is shortened by the presence of oxygen; therefore, the oxygen gradient profile can be estimated by observing fluorescence lifetime variation of the dye in the cell culture channel according to the Stern-Volmer equation. The oxygen gradients generated inside the cell culture channel are characterized by filling the oxygen sensitive fluorescence dye into the channel, and detected using frequency domain fluorescence lifetime imaging microscopy (FD-FLIM). The microscope is constructed based on an inverted fluorescence microscope (DMI 6000B, Leica Microsystems, Wetzlar, Germany) equipped with a modulated light emitting diode (LED) light source (M470L3, Thorlabs, Inc., Newton, NJ, USA) with nominal wavelength of 470 nm, and a two tap CMOS FLIM camera (pco.flim, PCO AG, Kelhien, Germany) specifically designed for FLIM applications^20,28^. The detail characterization process is described in the Supplementary Information.

### Endothelial cell culture

In the cell experiments, human umbilical vein endothelial cells (HUVECs) (CC-2519, Lonza Group. Ltd., Basel, Switzerland) are cultured. The HUVECs are cultured using the growth medium kit (CC-3162, Lonza Group Ltd.), and the stock HUVECs are maintained in a humidified cell incubator at 37 °C with 5% CO_2_. The cells are passaged using 0.25% Trypsin-EDTA (Gibco 25200, Invitrogen Corp., Carlsbad, CA), and HUVECs with passage number of 4 to 6 are used for all the experiments performed in this paper for consistent experimental results. In order to prepare the device for the cell experiments, the entire device is first exposed to UV light for an hour for sterilization. The cell culture channel is then coated with an extra-cellular matrix (ECM) protein, fibronectin (FC010, EMD Millipore, Billerica, MA), with concentration of 100 µg/ml in Dulbecco’s phosphate-buffered saline (DPBS) (Gibco 14190, Invitrogen Corp.) at 37 °C for more than an hour before seeding the cells to promote cell adhesion. For cell seeding into the device, HUVECs with population of 4 × 10^5^ cells in 200 µl growth medium are introduced into the cell culture channel from the middle inlet. The cells are then cultured in the microfluidic device, placed in a cell incubator, for more than 24 hours to ensure that the cells well attach onto the substrate and reach confluence.

### Collective cell migration assay

To conduct the collective cell migration assay in the developed microfluidic device, cell patterns with well controlled sizes are first created using a laminar flow patterning method by taking advantage of the unique property of microfluidics^29^. In the microfluidic collective cell migration assay, the cell patterns are generated at the middle of the confluent cell layer. The migrations of the cells toward two sides under different conditions are then observed. The formed cell patterns mimicking cell arrangements during angiogenesis enable study of endothelial cell proliferation and migration in various physiological activities. In the cell pattern formation process, the growth medium is first introduced into the device from the middle inlet of the cell culture channel with flow rate of 20 µl/min, and then the trypsin solutions are injected into the device from the two side inlets of the middle channel with flow rates of 10 µl/min for more than 3 minutes as shown in Fig. 1(d). The fluidic actuation with well-controlled flow rates is achieved using syringe pumps. The cell pattern formation within the channel is real-time monitored under an inverted fluorescence microscope (AF7000, Leica Microsystems, Wetzlar, Germany). The trypsin solutions are stopped when observing the detachment of HUVECs from the substrate, and the flow rate of the growth medium is increased to 1 ml/min for a minute to wash out the excessive trypsin and the HUVECs partially attaching onto the substrate. The tubings and interconnections for the cell patterning are then detached from the device, and reservoirs are connected to the inlet and outlet of the cell culture channel to supply medium for the collective cell migration assay experiments. Growth medium containing 100 µg/ml fibronectin is first flowed into the cell culture channel, and incubated at 37 °C for an hour to assure the proper surface conditions for cell migration and proliferation. Finally, the medium inside the cell culture channel is replaced by the normal growth medium and incubates for more than 30 minutes before the collective cell migration assay experiments.

To compare the endothelial cell collective migration under different culture oxygen conditions, the collective cell migration assays under three conditions are tested: normoxia (~20% O_2_), hypoxia (1% O_2_), and oxygen gradients. The uniform normoxia and hypoxia environments are controlled using a cell incubator (HERACell 240i, Thermo Fisher Scientific, Inc., Waltham, MA, USA) in the experiments. The oxygen tension in the incubator is lowered by flowing pure nitrogen gas into the incubator. Furthermore, three different medium compositions including: normal complete cell culture medium, the medium with 0.1 µM cytochalasin-D, and the medium with 25 µg/ml YC-1 are also exploited to study effects of the drugs on the collective cell migration under the different oxygen conditions. The drugs, cytochalasin-D and YC-1, are exploited to study effects of cell cytoskeleton disruption and HIF inhibition on endothelial cell proliferation and migration under various oxygen conditions, respectively. Titration of the drugs is performed to estimate the cell viabilities under different concentrations and two oxygen tensions (normoxia and 1% O_2_ hypoxia), and the detail process and results are described in the Supplementary Information. The concentrations around IC50 of both drugs are selected for the cell experiments.

During the collective cell migration assay experiments, the entire setup including: the microfluidic device and the syringe pumps, is placed inside the 37 °C and 5% CO_2_ humidified cell incubator with oxygen concentration controllability. The oxygen concentration of the incubator is set as 20% for normoxia and oxygen gradient experiments, and 1% for hypoxia experiments. In the experiments with oxygen gradients, the chemical reactants, 100 mg/ml pyrogallol and 1M NaOH solutions, for the oxygen scavenging reaction are introduced into the chemical reaction channel with flow rates of 10 µl/min from the different inlets.

In order to eliminate effect of shear stress on HUVECs in this study, the growth medium is kept static in the cell culture channel by filling the medium reservoir with similar heights during the observation. The images of the cells inside the channels are either recorded using a live cell analyzer (JULI, NanoEnTek, Seoul, Korea) in real time (an image is captured every 3 minutes) inside the cell incubator, or using the inverted microscope right before the experiments and at the 8th and 16th hours. The images are then analyzed using an image processing and analysis software, ImageJ (Ver. 1.46, National Institute of Health, Bethesda, MD) for collective cell migration speed estimation. In the analysis, decreases of areas without the cells on both sides during the experiments are measured, and the collective cell migration speeds are calculated by normalizing the area reduction to the channel length and the time period. For each device, the migration speeds in upstream, midstream, and down-stream areas are characterized, and all the experiments are repeated three times for statistical analysis.

### Assay data analysis

#### Collective cell migration speed analysis

**Figure 2:**
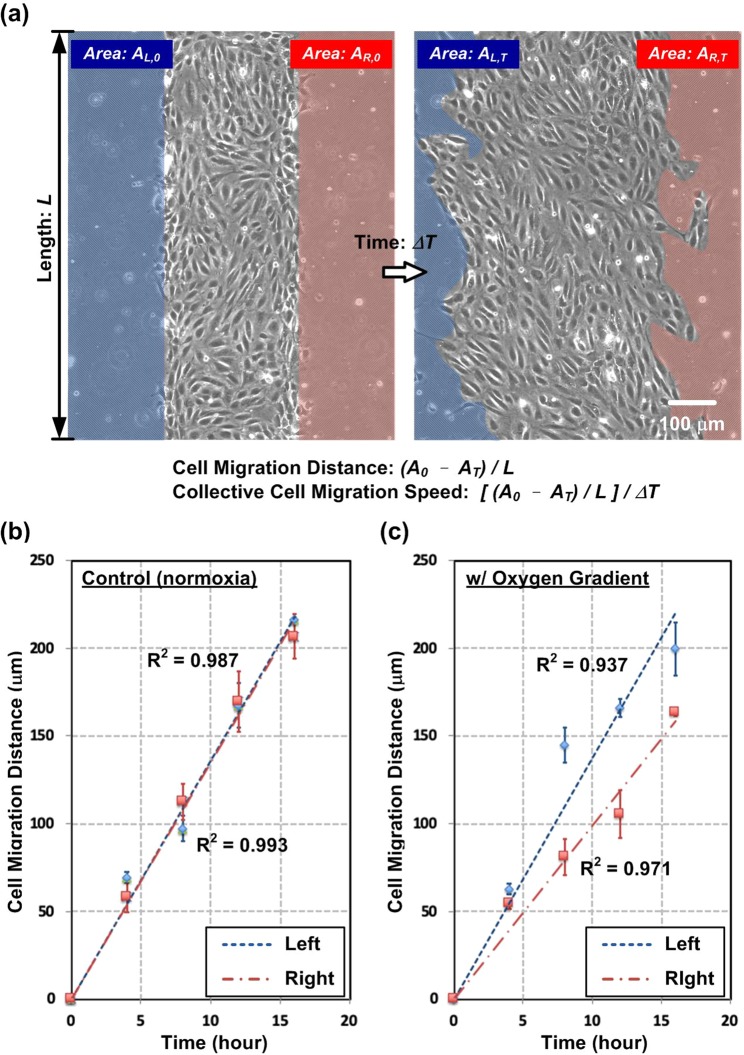
(**a**) Analysis of speed of the collective cell migration performed within the microfluidic device. (**b**,**c**) Cell migration distance variation of HUVECs cultured in the microfluidic devices using growth medium with and without oxygen gradient (n = 3).

In order to quantify the collective cell migration speed, linear regressions are exploited to fit the migration distances during the time period measured in the experiments. Figure 2 shows the analysis procedure and the examples of the migration speed analysis of the HUVECs cultured in the device. The cell migration distances are estimated by analyzing the captured microscopic images at different time points as shown in Fig. 2(a). The collective cell migration speed is then calculated by normalizing the cell migration distance to the time period using the equations as shown in Fig. 2(a). In order to further consistently investigate the collective cell migration speed of HUVECs under various oxygen conditions; the migration speeds are normalized to that of HUVECs cultured under the control condition (with growth medium under normoxia) performed in the experiments at the same time.

#### Migration directionality analysis

To quantitatively analysis the directionality of the collective cell migration speed, migration directionality (*D*) defined as

$$D=({S}_{R}-{S}_{L})/({S}_{R}+{S}_{L})$$

is calculated for all the experiments. In the equation, *S*_*R*_ and *S*_*L*_ are the collective cell migration speeds toward right (higher oxygen tension) and left (lower oxygen tension), respectively. The calculated directionality is a value between 1 and −1. When the migration speeds toward left and right are similar, the directionality is closed to zero. If one of the migration speeds is much faster than another, the directionality approaches 1 (toward right) or −1 (toward left).

#### Cell proliferation analysis

Since both proliferation and migration of the cells are involved in the collective cell migration assay^30^, the collective cell migration speed of the HUVECs highly depends on their proliferation rate and/or migration speed. In order to investigate the effect of cell proliferation on the collective cell migration, the cell numbers before and after the 16-hour assays are counted based on the brightfield phase images for all the experimental conditions. The normalized cell number is then calculated by dividing the cell number after the experiment by that before the same experiment. Therefore, the larger normalized cell number refers to the higher proliferation rate of the cells.

## Results and Discussion

### Oxygen gradient characterization

**Figure 3:**
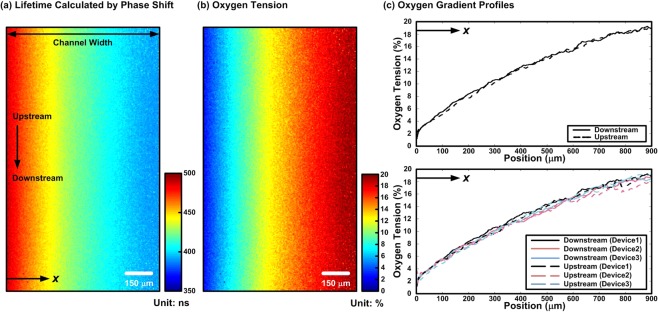
(**a**) Fluorescence lifetime image measured using FD-FLIM system based on fluorescence signal phase shift. (**b**) Oxygen tension profile calculated based on the measured fluorescence lifetime and Stern-Volmer equation. (**c**) Oxygen gradient along the channel width measured at up- and down-stream of the cell culture channel, and comparison of the oxygen gradients generated in three different devices.

The oxygen gradients generated inside the cell culture channel are characterized using the FD-FLIM, and the results are shown in Fig. 3. Figure 3(a) shows the experimentally measured fluorescence lifetime of the oxygen sensitive dye across the width of the cell culture channel when performing the oxygen scavenging chemical reaction in the neighboring chemical reaction channel. The spatial oxygen tension profile can then be calculated according to the Stern-Volmer equation as shown in Fig. 3(b). In order to confirm the consistency of the oxygen gradient profiles along the channel, oxygen gradients at the upstream (5 mm up from the middle of the channel along the flow direction) and at the down stream (5 mm down from the middle of the channel along the flow direction) are plotted for comparison. The resulted oxygen gradients across the cell culture channel width at up- and down-stream areas are plotted in Fig. 3(c). The results show that the consistent oxygen gradients with oxygen tensions ranging from approximately 1% to 19% across the 1 mm-wide cell culture channel can be reliably established in the microfluidic device, and no significant differences (less than 0.2% of oxygen tension) between the gradient generated in up- and down-stream of the cell culture channel. To further confirm the consistency of the oxygen gradients between different devices, the gradients within three different devices at up- and down-stream areas are also measured and shown in Fig. 3(c). The results also confirm the good consistency of the oxygen gradients generated using the spatially confined chemical reaction method between the devices.

### Cell pattern formation within microfluidic channel

**Figure 4:**
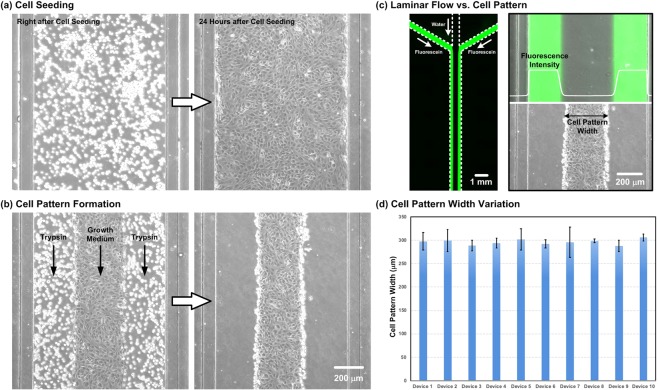
(**a**) Brightfield phase images of the HUVECs right after and 24 hours after seeding in the cell culture channel in the microfluidic device. (**b**) Phase images of the cell pattern formation on cultured HUVECs using laminar flow patterning. (**c**) Comparison of laminar flow pattern and the cell patterns formed within the cell culture channel. (**d**) Comparison of the widths of the cell patterns formed within ten different devices.

HUVECs are exploited to study endothelial cell proliferation and migration under various combinations of oxygen gradients, tensions, and drug treatments. Figure 4 shows the microscopic images of the HUVECs cultured in the cell culture channel on the microfluidic device. Figure 4(a) shows the brightfield phase images of the HUVECs right after seeding into the channel and after 24-hour culture in the device. The images show that the cells can well attach onto the fibronectin-coated substrate, and reach confluence after cultured in the device. Figure 4(b) shows the images during and after the laminar flow patterning for the cell pattern formation. The cell pattern width is also compared with the laminar flow pattern generated using fluorescein solution and water with the same flow conditions as shown in Fig. 4(c). The results demonstrate that the cell pattern can be successfully formed with precisely controlled dimensions that are similar to the laminar flow pattern using the method. The widths of the cell patterns formed in the center of the channels are approximately 300 µm and are consistent from the up- to down-stream within the cell culture channel. A video clip recorded during the collective cell migration process is shown in the Supplementary Information (Video 1). The cell pattern widths generated in ten different devices are also compared, and the results are plotted in Fig. 4(d). The results show that no statistical difference between the pattern widths for the ten different devices confirming the stability and consistency of the cell pattern formation process.

### Collective cell migration assays under various conditions

**Figure 5:**
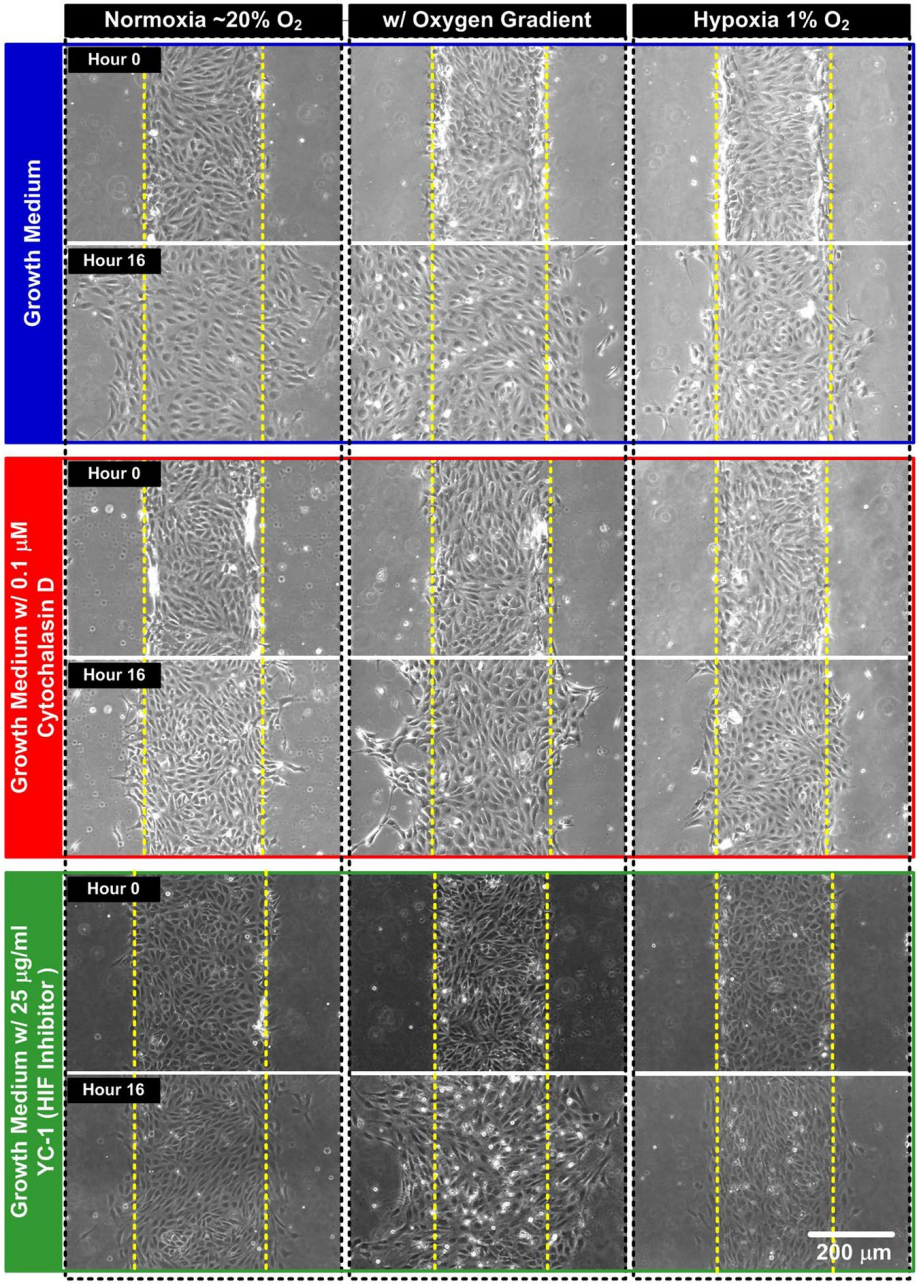
Brightfield phase images of the HUVECs before and after 16-hour collective cell migration assays performed within the microfluidic devices under various oxygen tensions, gradients, and drug treatments.

During the collective cell migration assay experiments, the microscopic images or videos are captured to investigate the cell proliferation and migration. Video clips recorded during the collective cell migration process under various conditions are shown in the Supplementary Information (Videos 2, 3 and 4). Figure 5 shows the microscopic brightfield phase images captured before the migration experiments and at 16th hour during the experiments with the cells cultured in different growth medium compositions under three different oxygen conditions: normoxia (~20% O_2_), oxygen gradient (1 to 19% O_2_), and hypoxia (1% O_2_). The experimental photos show that similar cell migration speed on both sides in the normoxia and hypoxia experiments due to the uniform oxygen tension distribution across the channel widths. In contrast, the photos suggest that the cell migration speed on the left side (toward lower oxygen tension) is faster than that on the right side (toward higher oxygen tension) of the cells cultured under oxygen gradients. Although the speeds are different for the cells treated with various growth medium compositions (cytochalasin-D and YC-1), the directional collective cell migration phenomena do exist for the HUVECs cultured under all three medium compositions.

For the quantification of the collective cell migration of the HUECs in the experiments, Fig. 2(b) shows the plot of the typical migration distances under normoxia culture conditions during the 16-hour experimental period. The plot shows closed slopes of the regression lines indicating similar collective cell migration speed on both sides under the uniform oxygen tension distributions. In contrast, Fig. 2(c) shows the different slopes of the regression lines for the experiments conducted under the oxygen gradient. The result suggests that the migration speed on the left side (larger slope toward lower oxygen tension) is faster than that on the right side (smaller slope toward higher oxygen tension). In the plots, the high coefficients of determination (R^2^) of the regressions suggest the nearly constant migration speed of the HUVECs during the experiments.

**Figure 6:**
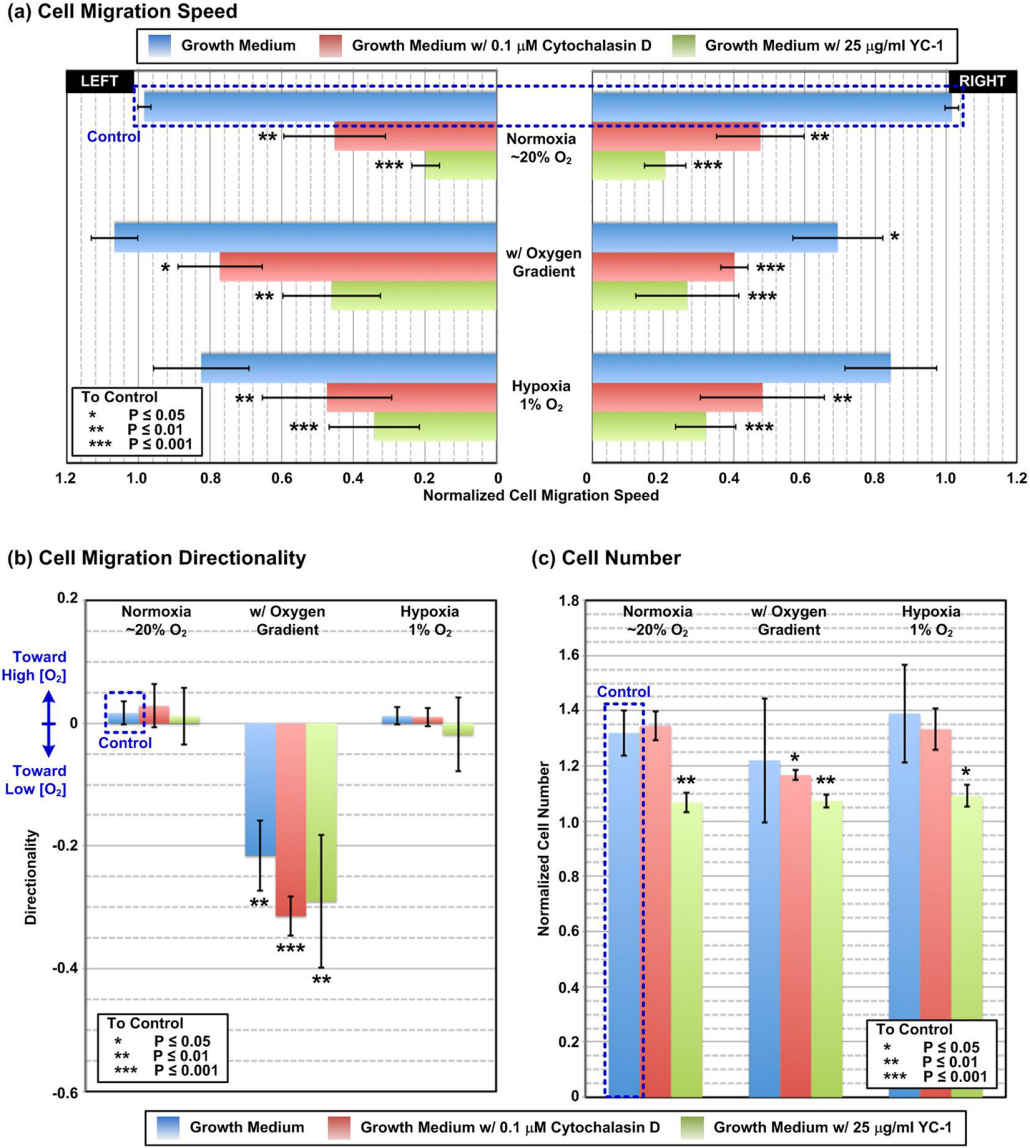
Quantitative analysis results of (**a**) collective cell migration speed; (**b**) cell migration directionality; and (**c**) cell number variation during the HUVEC collective cell migration assays under various combinations of oxygen tensions and gradients, and drug treatments (n = 3).

Figure 6 summarizes the collective cell migration assay data analysis results. Figure 6(a) shows the normalized HUVEC migration speeds toward left and right under various combinations of oxygen gradients, tensions, and drug treatments. To compare the difference between the normalized collective cell migration speeds under various experimental conditions to that in the control experiments, unpaired, two-tailed Student’s t-tests are performed for statistical analysis. The plot shows that the migration speeds are greatly reduced for the HUVECs cultured in the medium containing either cytochalasin-D or YC-1 for all the oxygen conditions. For the cells cultured in the uniform normoxia or hypoxia (1% O_2_), the speeds are reduced for more than 50% comparing to that in the control experiments. The results indicate that the drugs have great impact in regulating HUVEC collective cell migration process. Under uniform oxygen conditions, the migration speeds toward left and toward right are similar for all the medium compositions. In contrast, the migration speed toward left (higher oxygen tension) is higher than that toward right (lower oxygen tension) when the HUVECs are exposed to oxygen gradients regardless of the medium compositions. The directional migration results suggest that the oxygen gradients may play critical roles in endothelial cell collective migration, and the observation has never been discussed in the existing literature.

Figure 6(b) shows the calculated directionality under various conditions. Unpaired, two-tailed Student’s t-tests are also performed to statistically analyze the differences between the directionality obtained under different experimental conditions to the control one. All the migration directionality values calculated from the experiments conducted in uniform oxygen conditions (normoxia and 1% O_2_ hypoxia) are closed to zero (the absolute average values are no larger than 0.02), suggesting the similar migration speeds toward both directions. In contrast, the directionality values for the experiments conducted with oxygen gradients are negative and smaller than −0.21, which are significantly lower than those obtained from the uniform oxygen conditions. The negative values indicate that the migration speeds are faster toward left with lower oxygen tension. In addition, no significant difference between the directionality calculated from the cells cultured in different medium compositions under the oxygen gradients. The results suggest that disturbing cytoskeleton rearrangement (by cytochalasin-D) or inhibiting HIF related pathway (YC-1) has minimal effects on the directional collective cell migration of HUVECs under the oxygen gradients. Furthermore, the results also demonstrate that the oxygen gradient is a key factor guiding the directional collective cell migration process, and the drug treatments tested in the experiments have minimal effects on the directionality of the migration.

The calculated normalized cell numbers for all the experimental conditions are plotted in Fig. 6(c). In the analysis, unpaired, two-tailed Student’s t-tests are performed to statistically analyze the difference between the normalized cell numbers obtained under different experimental conditions to the control one. In all three different oxygen conditions, the cell numbers are similar when the HUVECs cultured in the normal growth medium and cytochalasin-D, except the cell number is slightly lower in the oxygen gradient experiments. Therefore, the slower collective cell migration speed of the cells cultured in the growth medium and cytochalasin-D in the experiments are mainly caused by the lower motility of the HUVECs. In comparison, the cell numbers are all lower than the control one for the HUVECs cultured in the HIF inhibitor, YC-1. As a result, the lower collective cell migration speeds for the YC-1 treated cells might be resulted from both lower proliferation and migration rates.

In addition, the normalized cell numbers obtained in the oxygen gradient experiments are closed to those in the uniform oxygen tension ones, indicating the similar cell proliferation rates of the cells treated with the same drugs cultured under different oxygen conditions. Therefore, the directional collective cell migration speed patterns shown in Fig. 6(b) are mainly caused by the directional migration of the HUVECs under the oxygen gradients. The results further emphasize the critical roles of spatial oxygen gradients in guiding the endothelial cell migration process during various physiological activities. The polarized cell migration behaviors under the presence of oxygen gradients have not been discussed in existing literature before, and require more detail study of the underlying mechanisms in the future. More importantly, the experimental results demonstrate that the microfluidic collective cell migration assay developed in this paper is capable of providing a practical yet powerful tool for *in vitro* cell culture studies that can be applied to various biomedical research topics.

## Conclusion

In this paper, a novel collective cell migration assay using a microfluidic device is developed. The device is capable of reliably generating cell patterns with consistent dimensions using laminar flow patterning for the assay. In addition, stable oxygen gradients can be generated for the assay in the device using the spatially confined chemical reaction method. The method eliminates utilization of bulk gas cylinders, sophisticated gas flow controller, and tedious interconnections allowing minimal chemical consumption and conventional cell incubator compatibility. The device is exploited to study endothelial cell proliferation and migration under combinations of three oxygen conditions (normoxia, oxygen gradient, and 1% hypoxia) and three medium compositions (growth medium, the medium with F-actin polymerization inhibitor, cytochalasin-D, and with HIF inhibitor, YC-1). The experimental results indicate that the collective cell migration speeds of the endothelial cells are faster toward to the lower oxygen tension sides regardless of the medium compositions due to their directional migration under the gradients. The observed directional migration behavior has never been discussed before, and can play critical roles in angiogenesis during various physiological activities. With the advantages and functionalities provided by the developed assay, more advanced studies on endothelial cells can be conducted to better understand their responses *in vivo* that are critical in a number of biomedical research fields.

## Supplementary information

Supplementary Information

Video 1 - Cell Pattern Formation

Video 2 - Collective Cell Migration (Control)

Video 3 - Collective Cel Migration (Oxygen Gradient)

Video 4 - Collective Cell Migration (Hypoxia)
