# Supplementary material for: Microfluidic Collective Cell Migration Assay for Study of Endothelial Cell Proliferation and Migration under Combinations of Oxygen Gradients, Tensions, and Drug Treatments
Source: Sci Rep. 2019 Jun 3;9:8234. doi: 10.1038/s41598-019-44594-5 (PMC6546762; doi:10.1038/s41598-019-44594-5)
Supplement: Supplementary file 1 — Supplementary Information [file 41598_2019_44594_MOESM1_ESM.docx]

**Supporting Information**

Microfluidic Collective Cell Migration Assay for Study of Endothelial Cell Proliferation and Migration under Combinations of Oxygen Gradients, Tensions, and Drug Treatments.

Hsiu-Chen Shih*^a^*, Tse-Ang Lee*^a^*, Hsiao-Mei Wu*^a^*, Ping-Liang Ko*^a^*, Wei-Hao Liao*^a^* and Yi-Chung Tung**^a,b^*

*^a^ Research Center for Applied Sciences, Academia Sinica, Taipei, 11529, Taiwan.*

*^b^ College of Engineering, Chang-Gung University, Taoyuan, 33302, Taiwan*.

** Corresponding author: Y.-C. Tung; TEL: +886-2-2787-3138; E-mail: tungy@gate.sinica.edu.tw*

**DRUG TITRATION**

In order to estimate the IC50 concentration of the drugs (cytochalasin-D and YC-1) tested on the HUVECs in the microfluidic collective cell migration assays, titrations of the drugs based on a cell viability assay are performed. In the experiments, the cell viability is evaluated using alamarBlue cell viability reagent (DAL1100, Invitrogen). The cell viability reagent is capable of detecting metabolically active cells and can be exploited for the quantitative analysis of cell viability and proliferation based on either absorbance or fluorescence-based measurement.

During the drug titration, 10,000 HUVECs dissociated in 100 μl of the growth medium are plated in 96-well plates and are allowed to adhere and grow for overnight in a humidified cell incubator at 37°C and 5% CO_2_ before proceeding with the drug treatments. The cells are then treated with cytochalasin-D or YC-1 by replacing the medium with that with different drug concentrations for 16 to 20 hours in an oxygen controlled cell incubator with normoxia (~20% O_2_) and hypoxia (1% O_2_) conditions. The amlamarBlue reagent with volume of 10 μl was then added in the medium after the drug treatments, and the cells are incubated for 4 hours at 37°C in the cell culture incubator for the reagent reaction. The plates are then measured the absorbance at wavelength of 570 nm and record results using a microplate reader (Synergy 2, BioTek Instruments, Inc., Winooski, VT). All the experiments are repeated three times for statistical analysis.

Figure S1(a) and (b) show the measured cell viability of the HUVECs treated with cytochalasin-D and YC-1 under the normoxia and hypoxia conditions, respectively. The viability is normalized to that obtained from the cells without the drug treatments for comparison. As can be seen in the results, the IC50s of the cytochalasin-D and YC-1 on HUVECs are approximately 0.1 μM and 25 μg/ml for both normoxa and hypoxia conditions, respectively.


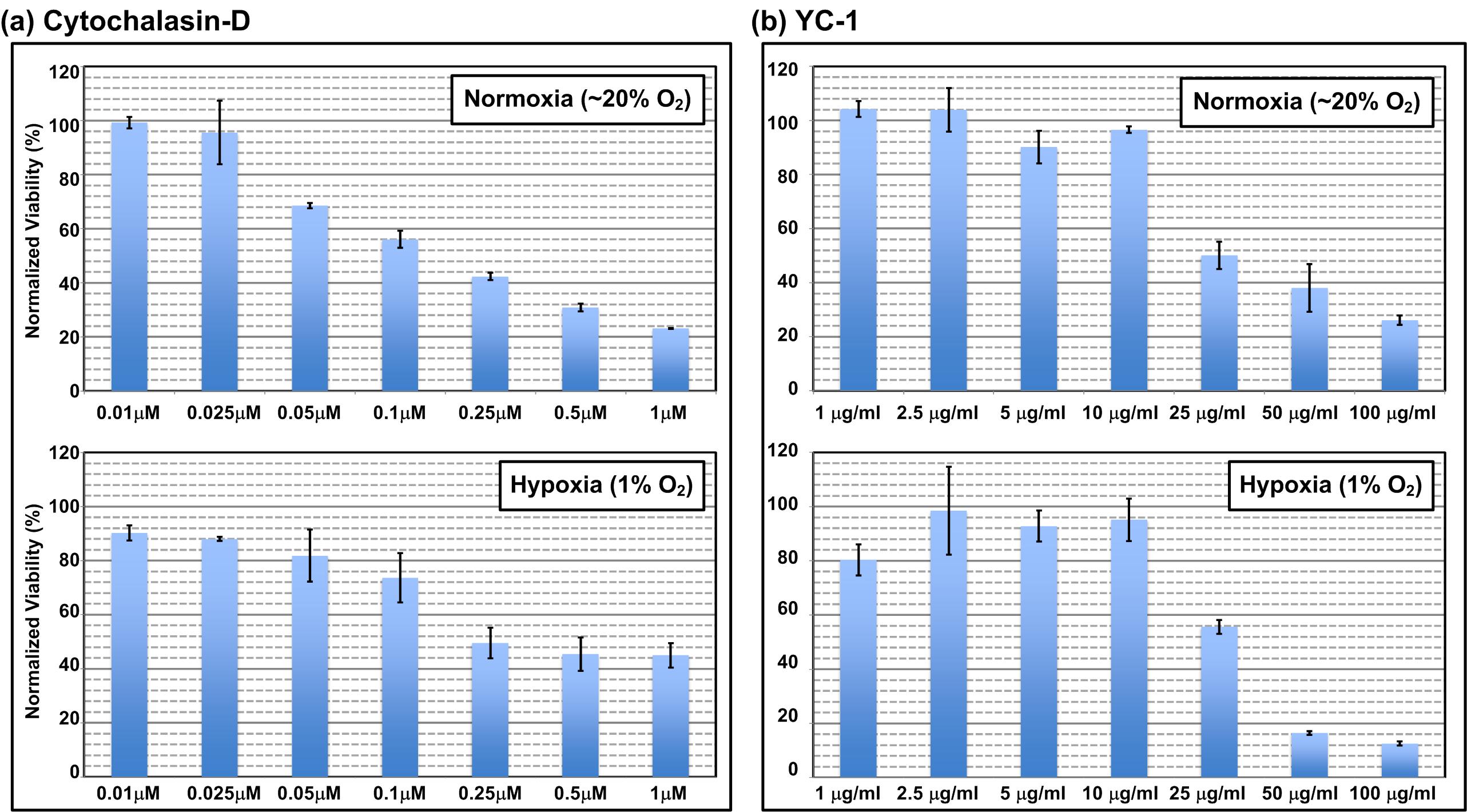


**Figure S1.** Normalized cell viabilities of the HUVECs treated with (a) cytochalasin-D and (b) YC-1 under normoxia and hypoxia conditions. The viability is estimated using alamarBlue cell viability reagent.

**FD-FLIM Oxygen Gradient Characterization**

In order to characterize the oxygen gradients generated in the microfluidic devices during the collective cell migration, a widefield frequency domain fluorescence imaging microscopy (FD-FLIM) setup based on a commercially available inverted fluorescence microscope (DMI 6000B, Leica Microsystems, Wetzlar, Germany) as shown in Figure S2 is exploited. The microscope is equipped with a high power LED with a nominal wavelength of 470 nm (M470LP-C2, Thorlabs, Newton, NJ) as a fluorescence excitation light source, and a dual tap CMOS FLIM camera (PCO.FLIM, PCO AG, Germany) as an imaging sensor. The LED and the camera are synchronized and modulated with a 250 kHz digital signal generated by the camera. The FD-FLIM setup provides rapid and accurate oxygen gradient measurement capability without tedious calibration processes and sophisticated instrumentation.

For oxygen gradient measurements, an oxygen sensitive fluorescent dye, tris(2,2’-bipyridyl) ruthenium(II) chloride hexahydrate (RTDP; 50525-27-4, Acros Organics, Geel, Belgium), with concentration of 5 mg/ml is introduced into the cell culture channel on the microfluidic device. The fluorescence lifetime of the dye can be shortened with the presence of oxygen, and the oxygen concentration can be estimated from the lifetime variation according to Stern-Volmer equation:

$$\frac{\tau_{0}}{\tau}=1+K_{q}\times[O_{2}]$$

where *τ_0_* and *τ* are the fluorescence lifetimes without and with the presence of oxygen, respectively, and *K_q_* is a quenching constant. The quenching constant is calculated by measuring lifetime of drops of the aforementioned dye bubbled with pure nitrogen or oxygen gas (0.1 psi for 8 minutes) as standard samples for depleted (0%) or saturated (100%) oxygen conditions sandwiched between glass slides. The lifetime of a drop of the dye in ambient environment (20.9% O_2_) with a known lifetime (*τ* = 381 ns) is utilized as a reference.^S1^ As a result, the lifetime distribution and the estimated oxygen gradient profiles can be estimated as shown in Figure 2. The detail characterization and analysis process are described in previous literature.^S2, S3^


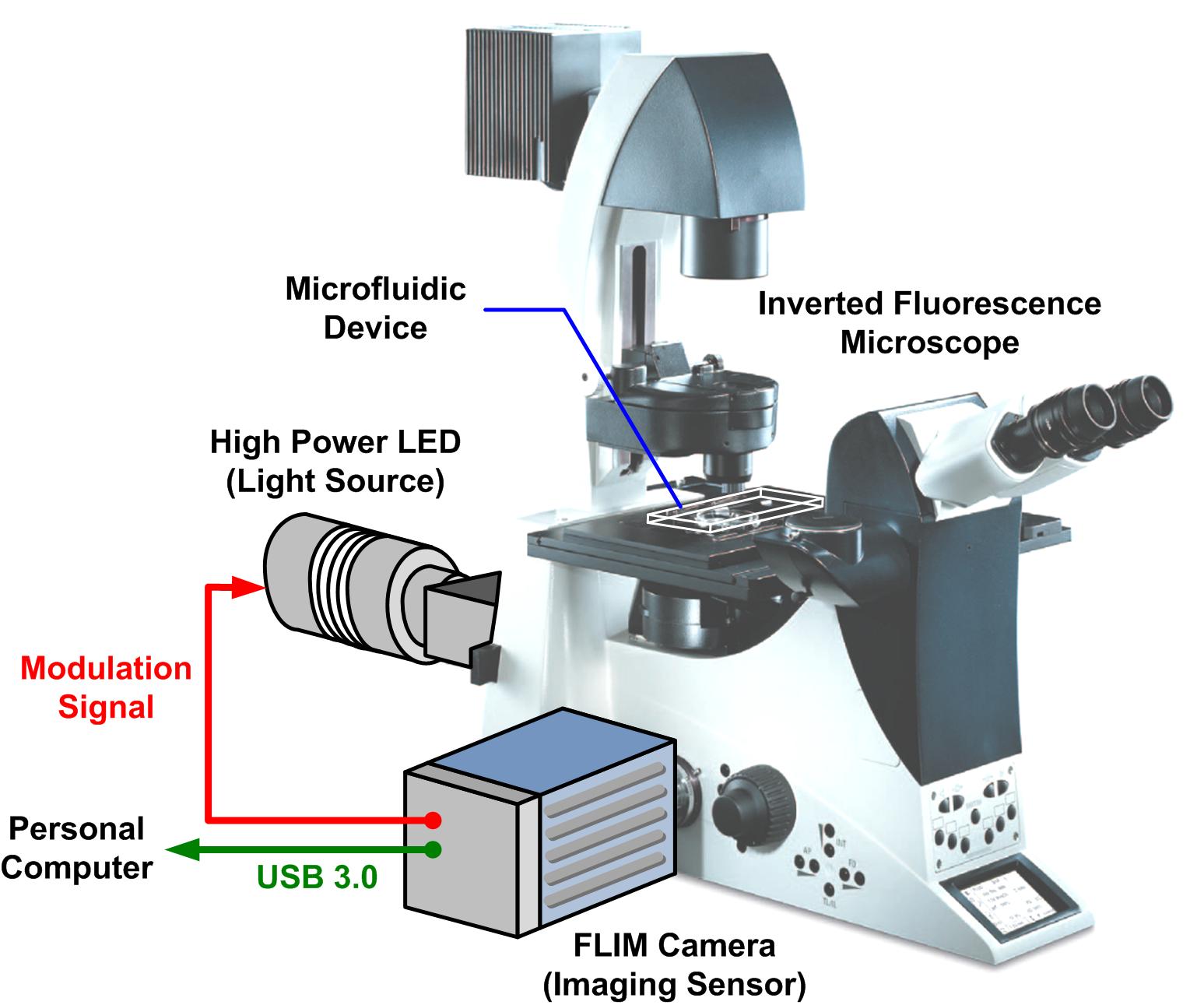


**Figure S2.** Experimental setup for the FD-FLIM oxygen measurements based on an inverted fluorescence microscope equipped with a high power LED light source and a FLIM camera.

**Description of Videos**

**Video1:** Cell pattern formation by laminar flow patterning in the microfluidic device. The video playback speed is 10X.

**Video2, 3, and 4:** Collective cell migration of HUVECs in the microfluidic devices cultured in normal growth medium with normoxia, oxygen gradient, and hypoxia (1% O_2_) conditions, respectively. The entire videos are for 16-hour experimental periods.

**REFERENCES**

S1 Zhong, W., Urayama, P. and Mycek, M. A. *J. Phys. D* **2003**, 36, 1689-1695.

S2 Wu, H. M., Lee, T. A., Ko, P. L., Liao, W. H., and Tung, Y. C. *22nd International Conference on Miniaturized Systems for Chemistry and Life Sciences*, 11-15, Kaohsiung, Taiwan. **2018**; 1196–1199.

S3 Wu, H. M., Lee, T. A., Ko. P. L., Liao, W. H., and Tung, Y. C. *Analyst* **2019**, DOI: 10.1039/C9AN00143C
